# Influence of Regular Statin Intake on Prostate‐Specific Antigen Values, Prostate Cancer Incidence and Overall Survival in a Prospective Screening Trial (ERSPC Aarau)

**DOI:** 10.1002/cam4.70485

**Published:** 2025-01-06

**Authors:** Alkiviadis Papagiannakis, Maciej Kwiatkowski, Stephen F. Wyler, Ashkan Mortezavi, Lukas Manka, Marian S. Wettstein, Rainer Grobholz, Angelika Hammerer‐Lercher, Daniel Eberli, Lukas Werner Prause

**Affiliations:** ^1^ Department of Urology Cantonal Hospital Aarau Aarau Switzerland; ^2^ Member of Medical Faculty University of Basel Basel Switzerland; ^3^ Department of Urology Academic Hospital Braunschweig Braunschweig Germany; ^4^ Department of Urology University Hospital Zurich Zurich Switzerland; ^5^ Department of Urology University Hospital Basel Basel Switzerland; ^6^ Department of Urology, Faculty of Health Sciences Brandenburg Brandenburg Medical School Theodor Fontane Potsdam Germany; ^7^ Department of Surgery, Division of Urology University of Toronto Toronto Canada; ^8^ Cantonal Hospital Aarau Institute of Pathology Aarau Switzerland; ^9^ Cantonal Hospital Aarau Institute of Laboratory Medicine Aarau Switzerland; ^10^ Member of Medical Faculty University of Bern Bern Switzerland

**Keywords:** chemoprevention, ERSPC, prostate cancer, prostate‐specific antigen, screening, statins

## Abstract

**Objective:**

While statins have demonstrated a variety of antineoplastic effects in preclinical studies, several retrospective clinical studies and observational studies have not shown a consistent chemopreventive benefit against prostate cancer (PCa). Therefore, in this population‐based cohort study, we examined the association of statin intake on prostate specific antigen (PSA) values and risk of development of PCa.

**Method:**

*N* = 4,314 men from the Swiss section of the European Randomized Study of Screening for Prostate Cancer (ERSPC) were evaluated. *N* = 761 men were statin users [Stat+]. The median follow‐up was 9.6 years. A transrectal prostate biopsy was performed in men with a PSA‐level ≥ 3 ng/mL. Mortality and incidence data was obtained through registry linkages. PCa incidence, total serum PSA level, free‐to‐total PSA level, and overall survival were compared between [Stat+] and [Stat−] patients.

**Results:**

Total PSA values were significantly lower in [Stat+] patients at baseline (1.5 vs. 1.8 ng/mL, *p* < 0.001) and at last follow‐up (1.8 vs. 2.1 ng/mL, *p* < 0.001). PCa detection during the follow‐up period was significantly associated with baseline PSA. The overall incidence of PCa showed no statistical difference among [Stat+] and [Stat−] groups (7.4% vs. 9.5%, *p* = 0.08), indicating that statin use had no effect on the risk of developing PCa during follow‐up. [Stat+] patients had a significantly higher overall mortality risk compared to [Stat−] patients (HR 2.04, *p* < 0.001).

**Discussion:**

A significant risk reduction in the development of PCa in [Stat+] patients was not found. We did observe lower PSA values among [Stat+] patients, compared to [Stat−] patients, with an increasing difference during follow‐up.

## Graphical abstract

Lower prostate‐specific antigen (PSA) values were found among statin users, compared to statin non‐users. A significant prostate cancer (PCa) risk reduction in statin users was not observed. Statin users had a significantly higher overall mortality risk than statin non‐users.

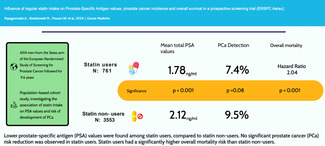

**Abbreviations**
ccm: cubic centimeter
CI: confidence interval
ERSPC: European Randomized Study of Screening for Prostate Cancer
f/t‐PSA: free/total prostate‐specific antigen ratio
HR: hazard ratio
IPSS: International Prostate Symptom Score
NSAID: non‐steroidal anti‐inflammatory drugs
PCa: prostate cancer
PSA: prostate‐specific antigen
SD: standard deviation
Stat+: statin user
Stat−: statin non‐user

## 1 Introduction

Prostate cancer (PCa) is the second most common cancer in men world‐wide, comprising 14.1% of all male cancers [1]. PCa is well known for its clinical, spatial, and morphological heterogeneity and molecular genetic diversity [2]. Differences in genetic background and epigenetic factors, such lifestyle, dietary factors, and medication use have been associated with variations in PCa risk [3, 4].

Statins are β‐Hydroxy β‐methylglutaryl‐coenzyme A (HMG‐CoA) reductase inhibitors that inhibit cholesterol synthesis [5]. Statins are a class of medications that have been implicated as affecting the development or clinical course of PCa and their use is an area of steadily increasing interest [4]. Their potential antineoplastic effect have been hypothesized to be linked to cholesterol‐mediated and non‐cholesterol‐mediated mechanisms affected by statins and related to key pathways involved in cancer formation and progression [5, 6].

Statins are widely used in the treatment of hyperlipidemia as part of the management of cardiovascular disease, making them among the most commonly prescribed medications worldwide. Statin use has been increasing, with 28% of US adults aged 40 and over reporting their use; simvastatin and atorvastatin are the two most frequently used statins [5, 6]. Data on statin use and the outcomes of elderly PCa patients taking these medications is often available.

Preclinical data demonstrate that statins possess anti‐inflammatory activity, inhibit angiogenesis and cellular proliferation, and promote apoptosis of PCa cells due to interference with cellular isoprenoid metabolism and prenylation‐dependent processes [6]. These activities all support a role for stains in the prevention or treatment of PCa. However, data from over 30 recent retrospective studies evaluating the association between statin use and the development of PCa have shown mixed results [6, 7, 8].

In order to examine the association of statin intake with serum prostate specific antigen (PSA) levels, the development of PCa, and overall patient survival we analyzed the Swiss (Aarau) section of the European Randomized Study of Screening for Prostate Cancer (ERSPC) [9, 10, 11].

## 2 Materials and Methods

### 2.1 Study Design and Recruitment of Participants

This study is a population‐based analysis of a cohort from the Swiss arm of the European Randomized Study of Screening for Prostate Cancer (ERSPC). The study protocol was performed as previously described [9, 10, 11]. In brief, it was a randomized population‐based trial investigating whether systematic PSA‐based screening reduces PCa mortality.

The study population is a cohort of 10,311 Swiss men, aged 55–70 years, recruited between September 1998 and August 2003. 5,159 were randomised towards screening arm and, during the initial screening round, 4,932 of these men participated. Continuous, daily use of statin was assessed from the second screening round onwards (hereafter defined as baseline). This study thus included 4,314 men who participated in the baseline screening. The third screening round took place 4 years after the baseline, until 75 years of age. Follow‐up data were reported at the time of the third screening round and patients were followed for a median of 9.6 years. The study protocol of the ERSPC requested a prostate biopsy in case of a PSA value ≥ 3.0 ng/mL [10]. All diagnoses of PCa were histologically confirmed by the Department of Pathology, University Hospital of Basel, Switzerland. Information on continuation of statin use was collected at the follow‐up visit. Data gathering was supervised by the ERSPC Epidemiology Committee, Pathology Committee, PSA Committee, Causes of Death Committee, an independent Data Monitoring Committee, and the Scientific Committee overlooking the study. Information on incidence and mortality was available through registry linkages up to September, 2014 [11].

### 2.2 Primary and Secondary Endpoints

The primary endpoint of our population‐based study was: (1) the association between statin use and PCa diagnosis and (2) the influence of statins on PSA levels at diagnosis and at follow‐up. Secondary endpoints were overall survival by statin group, PCa‐free survival by statin group, and risk stratification of PCa according to D'Amico criteria stratified by statin group [12].

### 2.3 Data Collection

Information was collected by questionnaire at each patient's visit to capture current medications, including statin therapy. Study nurse or physician in our clinic re‐confirmed the information given in the questionnaires. Information about maintenance of statin use was gathered at the follow‐up visit.

### 2.4 Statistical Analysis

Mean and standard deviation (SD) were calculated for continuous variables. Discrete measures were reported as number with relative frequency. Differences in baseline variables between statin users and nonusers were compared using the chi‐square test (categorical variables) and Mann–Whitney *U* test (continuous variables).

**FIGURE 1:**
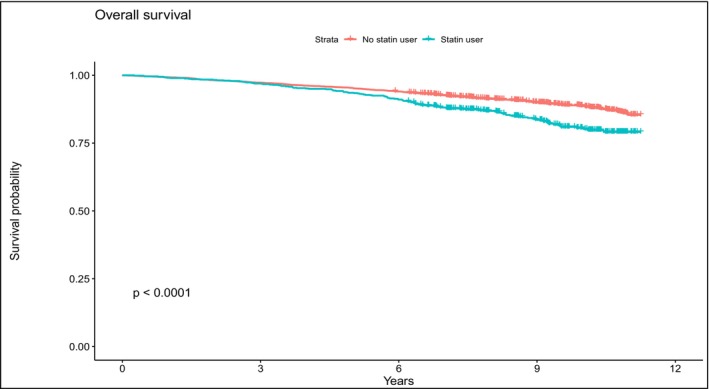
Kaplan–Meier estimates for overall survival of 4,314 men according to statin use (*p* < 0.0001, log‐rank test).

**FIGURE 2:**
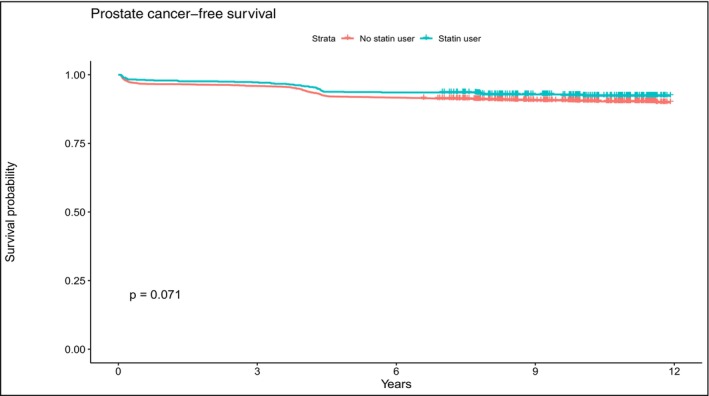
Kaplan–Meier estimates for prostate cancer‐free survival of 4,314 men according to statin use (*p* = 0.07, log‐rank test).

[Stat+] and [Stat−] group continuous variables were compared using the Welch two‐sample *t*‐test and discrete scaled variables were compared using the Pearson chi‐squared test. Univariate and multivariate Cox regression analysis was used to examine both the relationship between PCa detection and the all‐cause mortality with co‐variables of age, PSA, free‐to‐total PSA, family history of PCa, and statin use. Hazard ratios (HR) were estimated using Cox proportional hazard models with a 95% confidence interval (95% CI). Cox proportional hazards regression was used to calculate hazard ratios (as relative risks [RRs]) and 95% confidence intervals (CIs) of total PCa and of all‐ cause mortality (Table 2). Kaplan–Meier analyses were performed to evaluate overall survival (Figure 1) and PCa free survival (Figure 2) for both groups. The proportional hazard assumption was evaluated, and no violations were observed. Analyses were performed using IBM SPSS statistics 20 statistical software (Chicago, IL, USA), STATA version 12 (StataCorp LP, College Station, TX, USA), and R (R 3.5.2). All tests were two‐sided with a significance level set at *p* < 0.05.

### 2.5 Quality Control, Data Integrity and Ethical Approval

Quality control of data collection for the Swiss section of the ERSPC was conducted under supervision of the ERSPC. This study protocol of the Swiss arm of the ERSPC was approved by the ethical committee of northwest and central Switzerland (Record number 1998‐09‐09/RK). Written informed consent was obtained from each patient.

## 3 Results

### 3.1 Baseline and Follow‐Up Characteristics of Statin Users and Non‐Users

A total of 4314 men were evaluated and their mortality outcomes followed for a median of 9.6 years. 761 (17.6%) were taking a statin at baseline. Baseline and follow‐up characteristics of the study patients are presented in Table 1. Prevalence of a family history of PCa was similar between statin users and non‐users (Table 1). Patients using statin were 0.9 years older at baseline than [Stat−] patients (*p* < 0.001) (Table 1). No difference was observed in the D'Amico risk group profile of PCa statin users and non‐user patients at baseline and at follow‐up. Age at death was comparable in [Stat+] and [Stat−] men (Table 1).

**TABLE 1:** Comparison of unadjusted [Stat+] and [Stat−] patient baseline and follow‐up characteristics.

	Statin user (*N* = 761)	No statin use (*N* = 3553)	Overall (*N* = 4314)	
	*N* (%)	*N* (%)	*N* (%)	*p*
Age at baseline, (years)	66.3 (4.37)	65.4 (4.39)	65.5 (4.40)	**< 0.001**
Age at death during follow‐up, (years)	73.2 (5.53)	72.7 (5.56)	72.8 (5.55)	n.s.
PSA at baseline, [ng/ml]	1.54 (1.83)	1.80 (2.29)	1.75 (2.22)	**< 0.001**
f/t‐PSA ratio, baseline	29.3 (12.5)	27.0 (10.6)	27.4 (10.9)	**< 0.001**
PCa detection, baseline	16 (2.1%)	119 (3.3%)	135 (3.1%)	0.09
D'Amico PCa risk group, baseline				
Low risk	11 (1.4%)	83 (2.3%)	94 (2.2%)	0.26
Intermediate risk	5 (0.7%)	23 (0.6%)	28 (0.6%)	
High risk	0 (0%)	13 (0.4%)	13 (0.3%)	
PSA at follow‐up, ng/ml	1.78 (2.17)	2.12 (2.39)	2.06 (2.36)	**< 0.001**
f/t‐PSA Ratio, follow‐up	31.8 (49.4)	26.6 (12.0)	27.4 (22.4)	0.06
PCa detection, follow‐up	40 (5.3%)	217 (6.1%)	257 (6.0%)	0.42
D'Amico PCa risk group, follow‐up				
Low risk	18 (2.4%)	106 (3.0%)	124 (2.9%)	0.14
Intermediate risk	11 (1.4%)	77 (2.2%)	88 (2.0%)	
High risk	11 (1.4%)	32 (0.9%)	43 (1.0%)	
PCa detection, overall	56 (7.4%)	336 (9.5%)	392 (9.1%)	0.08
D'Amico PCa risk group, overall				
Low risk	29 (3.8%)	189 (5.3%)	218 (5.1%)	0.47
Intermediate risk	16 (2.1%)	100 (2.8%)	116 (2.7%)	
High risk	11 (1.4%)	45 (1.3%)	56 (1.3%)	

*Note:* *p* values which are deemed statistically significant are presented in bold. Follow‐up values are reported at the time of last follow‐up. PCa detection, follow‐up is the number of new cases of PCa detected during follow‐up. PCa detection, overall is the number of cases of PCa found at the time of last follow‐up. D'Amico PCa risk group are reported in a similar fashion. Values are given as mean (SD) or absolute (relative %) frequencies.

### 3.2 PSA And f/t‐PSA Ratio Over Time

Mean total PSA values were significantly lower among statin users, compared to non‐users at baseline and at last follow‐up (baseline: [Stat+] 1.54 ± 1.83 vs. [Stat−] 1.80 ± 2.29 ng/mL; *p* < 0.001; last follow‐up: [Stat+] 1.78 ± 2.17 vs. [Stat−] 2.12 ± 2.39 ng/mL; *p* = 0.001) (Table 1). [Stat+] men had a slightly elevated calculated free‐to‐total PSA ratio (f/t‐PSA) at baseline compared to [Stat−] men [Stat+] 29.3 ± 12.5 versus [Stat−] 27.0 ± 10.6; *p* < 0.001. The f/t‐PSA ratio of [Stat−] men and [Stat+] men at follow‐up was not different ([Stat+] 31.8 ± 49.4 versus [Stat−] 26.6 ± 12.0; *p* = 0.064).

### 3.3 PCa Detection Rate and Risk Factor Analysis

[Stat+] and [Stat−] patients had a similar incidence of PCa at baseline (*p* = 0.093) and at the follow‐up (*p* = 0.415) (Table 1). A PCa risk reduction was not observed at the time of follow‐up (Table 1). No significant difference was seen in the PCa free survival (*p* = 0.071) of the two groups (Figure 2). Statin users [Stat+] showed a higher overall mortality (*p* < 0.001) than [Stat−] patients (Table 2, Figure 1).

**TABLE 2:** Univariable and multivariable Cox regression proportional hazard analyses for prostate cancer detection and all‐cause mortality adjusted for age, use of statin, PSA level at baseline, free‐to‐total PSA and family history of PCa.

Characteristics	Univariable			Multivariable		
	HR	(95% CI)	*p*	HR	(95% CI)	*p*
Predicting prostate cancer detection during follow‐up						
Age, years	1.03	(1.01–1.05)	**0.006**	1.00	(0.97–1.02)	0.79
+ Statin use, baseline	0.771	(0.58–1.02)	0.07	0.943	(0.70–1.27)	0.70
PSA, baseline ng/ml	1.12	(1.10–1.13)	**< 0.001**	1.201	(1.17–1.23)	**< 0.001**
Free‐to‐total PSA	1.39	(1.22–1.58)	**< 0.001**	0.664	(0.54–0.81)	**< 0.001**
Family history	1.20	(0.81–1.78)	0.35	1.071	(0.71–1.61)	0.74
	# events = 392, *p*‐value (log‐rank test) = 0.063			# events = 364, *p*‐value (log‐rank test) < 0.001		
Predicting all‐cause mortality during follow‐up						
Age, years	1.10	(1.08–1.13)	**< 0.001**	1.12	(1.09–1.15)	**< 0.001**
+ Statin use, baseline	1.67	(1.36–2.04)	**< 0.001**	2.041	(1.55–2.69)	**< 0.001**
PSA, baseline, ng/ml	1.01	(0.98–1.05)	0.44	0.998	(0.94–1.06)	0.94
Free‐to‐total PSA, baseline	1.21	(1.02–1.45)	0.03	1.222	(0.93–1.60)	0.14
Family history	0.97	(0.67–1.40)	0.88	0.833	(0.50–1.40)	0.49
	# events = 500, *p*‐value (log‐rank test) < 0.001			# events = 268, *p*‐value (log‐rank test) < 0.001		

*Note: p* values which are deemed statistically significant are presented in bold.

Abbreviations: CI, confidence interval, HR, hazard ratio.

PCa detection during follow‐up was significantly associated with baseline PSA (multivariable Cox regression analysis, HR = 1.20; 95% CI, 1.17–1.23; *p* < 0.001), and baseline free‐to‐total PSA levels (multivariable Cox regression analysis, HR = 0.66; 95% CI, 0.54–0.81; *p* < 0.001) (Table 2). Higher baseline PSA values were associated with an increased risk of PCa detection during follow‐up (Table 2). Higher free‐to‐total PSA levels were associated with a decreased risk of PCa detection during the follow‐up period (Table 2). Statin use at baseline was not associated with the risk of developing PCa during the study period (HR 0.94, 95% CI 0.70–1.27; *p* = 0.70).

Statin use was associated with an increased risk of all cause death during the study period (multivariable Cox regression analysis, HR = 2.04; 95% CI 1.55–2.69; *p* < 0.001) (Table 2). Baseline free‐to‐total PSA ratio, baseline PSA values, and baseline family history of PCa were not associated with all‐cause mortality during follow‐up (Table 2).

## 4 Discussion

The risk of developing PCa in patients using statins has been a source of great interest, largely due to the epidemiology of PCa making it one of the most prevalent cancers in men, as well as the widespread use of statins making them one of the most common outpatient prescription medication classes. To date, mixed results have been reported about the association of statin use with PCa [13]. Our subgroup analysis of a cohort from the population‐based Swiss arm of the ERSPC evaluated the effect of statins on PSA values and the overall relative risk of PCa in patients systematically screened for PCa and followed for a median of 9.6 years [10]. According to the existing literature, this is the second study, after the Finnish Randomized Study of Prostate Cancer Screening, to analyze the outcome of a randomized PSA‐based screening intervention in men using statins.

### 4.1 Total Serum PSA Values and f/t‐PSA Ratio

Statins vary in molecular structure, binding affinity to HMG‐CoA reductase, requirements for enzymatic conversion to become active, bioavailability, and peripheral tissue concentration, which may lead to different effects on PSA levels and prostate metabolism. Together, these findings may explain why total PSA values were significantly lower in [Stat+] patients at the baseline evaluation and at follow‐up. Baseline PSA values were associated with the development of PCa during follow‐up, supporting the use of PSA in men taking statins who decide to screen for PCa. Observation of lower PSA levels among statin users have been documented in several studies [14, 15, 16, 17]. These findings suggest different statins may have different activities outside their main function as a lipid‐lowering medication and may explain the decreased PSA levels found in men taking statins, especially when they are used for more than 2 years [18]. However, one randomized controlled trial of statins and benign prostatic hyperplasia found no influence of statins on PSA levels.

HMG‐CoA reductase inhibitors affect the rate‐limiting step in cholesterol synthesis, the conversion of HMG‐CoA to mevalonate [18]. Intracellular changes in cholesterol metabolism have been shown to have multiple effects on cholesterol‐rich cellular membrane domains along with downregulating androgen and estrogen receptors [18].

Thus, the small number of prior studies suggests that statin drug use and cholesterol concentration either has no influence or may cause a small decrease in circulating PSA concentration.

### 4.2 Influence of Statins on the Incidence of PCa

A previous meta‐analysis showed that statin use was associated with a highly heterogeneous effect on PCa (relative risks: 0.26–2.94) [13]. Published meta‐analyses evaluating statin use in PCa patients have several limitations. There is a lack of randomized controlled trials evaluating the effect of statins on the natural history of PCa and its treatment. Published studies often do not control well for comorbidities in the [Stat+] and [Stat−] groups and [Stat+] patients in some publications were more likely to receive more intensive medical care and use more medical resources. Published studies do not document statin intake or account for type and dose of statin used.

A recent systematic review of the literature and meta‐analysis of 27 studies evaluating the effect of statin use on biochemical recurrence of PCa after radical prostatectomy or radiation therapy with curative intent showed that statin use before or after treatment did not improve the rate of biochemical recurrence [19].

In our study, [Stat+] and [Stat−] patients had a similar rate of PCa at baseline, of developing cancer during the study period, and of PCa at the time of follow‐up. These findings are consistent with those reported in previous meta‐analyses evaluating this effect. The use of statins did not appear to have an association on the development of PCa in our patients.

A post‐ hoc analysis study from the Finnish arm of the ERSPC evaluated 23,320 men from the general population, similar to our study. Patients in this arm were found to have a dose‐dependent decreased incidence of PCa associated with statin use, compared to non‐users [20]. There are several differences in the study populations. Patients followed in the Swiss section of the ERSPC had a different age range at enrolment, higher baseline PSA, and different rates of PCa development during follow‐up ([Stat+] patients, Aarau arm 5.3% vs. Finnish arm 4.0% [*p* = 0.10, chi squared test]; [Stat−] patients, Aarau arm 6.1% vs. Finnish arm 8.0% [*p* = 0.0001, chi squared test]). The relatively higher diagnosis rate in [Stat−] patients during follow‐up could contribute to the different findings in the two studies. Another possible confounding factor is the study size. Our study accrued about one‐tenth of the number of patients compared to the Finnish report. Our negative, but statistically borderline finding of no difference in the PCa free survival by statin group (*p* = 0.07, Figure 2) might have reached statistical significance if a larger number of patients had been evaluated.

### 4.3 Specifics of Statin Use

#### 4.3.1 Type of Statins Used

There is little data evaluating the effect of statin type, statin dosage, and duration of statin intake on the development or recurrence of PCa. Any potential statin chemopreventive results caused by statin interfering with lipid metabolism could be affected by the lipophilicity of different statins [21, 22] and several studies have concluded that the lipophilic statins might achieve better chemopreventive effects [15, 16, 17, 18]. In our study, we did not perform a subgroup analysis based on statin type. However, the predominantly prescribed statin in Switzerland during the study period is atorvastatin, which is a lipophilic statin.

#### 4.3.2 Duration of Statin Use and Outcomes

Serum PSA has been shown to be reduced in men taking statins for at least 6 months, and this effect is greatest after 2 years of statin use [14, 20]. The median follow‐up of patients was 9.6 years, this being comparable or greater than most previous reports. This long follow‐up suggests that relevant PSA changes should be well evolved in our patients.

Statin intake for 5 or more years has been reported to be associated with a protective effect against the development of PCa [18]. In our study population, we were not able to verify this protective effect. Although we could not report the exact duration of statin intake, we can reasonably infer that our study participants engaged in a minimum of 4 years of continuous statin usage between baseline assessment and the follow‐up visit.

#### 4.3.3 Statin Dose

A literature review and meta‐analysis evaluating the duration of statin use and cumulative defined daily dose of statin on the development of PCa did detect a benefit with statin use, however, it could not distinguish if the benefit was from long duration or large dose [23]. We did not perform a subanalysis of the different statin doses.

### 4.4 D'Amico Score

D'Amico Score has been used to define risk categories for PCa growth and spread [12]. Several studies have shown statin use is associated with a selectively lower risk of advanced PCa, where lower risk was defined using varying levels of Gleason grade, clinical stage or a combination of both variables [6, 24, 25, 26]. [Stat+] and [Stat−] patients we diagnosed had no difference in their D'Amico risk group profile at baseline and at follow‐up. Statins thus did not appear to affect the PCa risk profile of patients we treated over time. The relatively smaller number of patients treated here may have inhibited the ability to detect these differences.

### 4.5 Overall Mortality Risk

Statin use at baseline was a significant risk factor for predicting all‐cause mortality, but not for predicting PCa incidence during follow‐up. The statin users in our cohort were about 1 year older than non‐statin users at baseline. These findings are similar to previous retrospective reports where statin users tended to be older, more obese, more likely to have comorbid disease, and more commonly use NSAIDs (non‐steroidal anti‐inflammatory drugs), 5‐α reductase inhibitors, and α‐blockers, compared to those not using statins [18, 27, 28]. While PSA findings were associated with the development of PCa, they were not associated with all‐cause mortality during follow‐up. Thus, our findings may be attributed to an increased risk of death from other co‐morbidities that statin users likely have.

### 4.6 Limitations

One should consider several limitations when interpreting our findings. Our data may not be relevant to other patient populations with a different genetic or environmental background. The ERSPC is a prospective cohort study primarily designed to demonstrate the PCa‐related mortality reduction and use of this database to analyze different subgroups in a retrospective fashion is less significant concerning statements on other hypotheses. While the study did feature a population‐based and randomized design, which should reduce selection bias, a “healthy user bias” cannot be excluded [29].

Although we were able to identify if statins were prescribed, data regarding the types of statins used and prescribed doses were unavailable for evaluation, thereby precluding an analysis of these important factors. On the other hand, we were able to evaluate the patient's adherence which can be considered as high, given the fact that statin users had taken statins for at least 4 years. Interestingly, while the American College of Cardiology often references duration of 2 years or more in their guidelines when discussing statin therapy impact on cardiovascular outcomes, the duration of statin therapy to potentially achieve chemopreventive effects remains unclear [30].

As the study baseline was set at the second screening round, and thus 4 years after beginning of actual ERSPC screening trial there might be concern regarding immortal‐time bias exposure due to this 4 years delay interval. [31] However its impact on our study's findings can be considered negligible given the fact that during this period, only 82 participants in the screening cohort died, corresponding to a mortality rate of 1.6%. Regarding PCa‐specific mortality, a direct comparison is statistically not feasible, due to very small number of events during follow‐up [32]. This low number is likely attributed to the relatively short observation period, which may not capture the long natural history of PCa, compounded by the fact that most PCa‐related deaths occurred among individuals diagnosed during the first screening round. The effect of treatments for comorbidities which could adversely affect the measured outcomes, such as the administration of 5‐α reductase inhibitors, metformin, and aspirin, was not evaluated. Previous analyses of this group of patients revealed that the oral antidiabetic drug metformin had no significant effect on PSA levels, PCa incidence, or PCa grade, but was associated with a significantly higher risk of all‐cause mortality [22]. Since robust data are not yet available on these drugs, we did not adjust for these variables.

Additionally, consistent with most observational studies, our study lacked detailed data on participants' comorbidities and lifestyle factors, such as smoking, physical activity, and diet. The absence of this information may contribute to further confounding, as these unmeasured variables could potentially influence the observed associations between statin use and both overall mortality and PCa outcomes, either amplifying or attenuating the effect. To further explore the significance of statins in an organized versus opportunistic screening program the analysis of both screening arms per allocation of their randomization would be of advantage. This approach was unfortunately limited due to unavailable data for the control group. The impact of patients who declined a prostate biopsy is not known. Decreased PSA values due to statin intake could influence biopsy rates [18] and the ability to diagnose PCa at an early stage. Our findings are derived from a screening cohort with a mean age of 65.5 years and might therefore not be comparable to other populations with other age distributions.

## 5 Conclusions

With many observational studies supporting the hypothesis that statins may have a chemopreventive effect [14, 15, 16], our findings contribute further to the literature. In our large population‐based cohort, we found no association between statin use and PCa development. On the other side, PSA levels in statin users were significantly lower than in non‐users at baseline and at the follow‐up. Lower PSA values may imply a protective long‐term effect of statins on the development of PCa. It is noteworthy to mention that our study was conducted within an organized PCa screening trial and the observed reduction in PSA levels in the statin population could potentially impede the early diagnosis of PCa both in such a program and in real‐world settings where opportunistic screening is prevailing. Thus, our findings inform the future PCa screening health policy. While our results do not support the use of statins in the management of PCa, it is essential to interpret these findings considering several limitations inherent in our study. Notably, the lack of data on statin type and dosage, as well as the failure to control for significant confounding factors limit the conclusiveness of our findings. Therefore, we advocate for further long‐term, prospective randomized trials that are rigorously controlled for these confounding variables to accurately assess the effects of different statins on PCa outcomes.

## Author Contributions

**Alkiviadis Papagiannakis:** conceptualization (lead), data curation (lead), formal analysis (lead), funding acquisition (supporting), investigation (lead), methodology (supporting), project administration (lead), resources (supporting), software (supporting), supervision (lead), validation (lead), visualization (equal), writing – original draft (lead), writing – review and editing (lead). **Maciej Kwiatkowski:** conceptualization (lead), data curation (lead), formal analysis (lead), funding acquisition (lead), investigation (lead), methodology (lead), project administration (lead), resources (lead), software (equal), supervision (lead), validation (lead), visualization (equal), writing – original draft (supporting), writing – review and editing (lead). **Stephen F. Wyler:** investigation (supporting), supervision (supporting). **Ashkan Mortezavi:** validation (supporting). **Lukas Manka:** validation (supporting). **Marian S. Wettstein:** data curation (supporting), formal analysis (supporting). **Rainer Grobholz:** data curation (supporting). **Angelika Hammerer‐Lercher:** data curation (supporting). **Daniel Eberli:** supervision (supporting). **Lukas Werner Prause:** conceptualization (lead), data curation (lead), formal analysis (lead), investigation (supporting), methodology (lead), project administration (lead), resources (lead), software (supporting), supervision (lead), validation (equal), writing – original draft (lead), writing – review and editing (supporting).

## Conflicts of Interest

The authors declare no conflicts of interest.

## Data Availability

The data used in this study are available upon request from the corresponding author, following irreversible anonymization and in accordance with Swiss law regulations.
